# Supplementary material for: Changes in adenosine receptors and neurotrophic factors in the SOD1G93A mouse model of amyotrophic lateral sclerosis: Modulation by chronic caffeine
Source: PLoS One. 2022 Dec 14;17(12):e0272104. doi: 10.1371/journal.pone.0272104 (PMC9749988; doi:10.1371/journal.pone.0272104)
Supplement: S1 Table — (DOCX) [file pone.0272104.s001.docx]

| **Gene** | **Accession number** | **Primer sequence (5′–3′)** |
| --- | --- | --- |
| **A_1_R** | NM_001008533.3 | **Forward:** TCG GCT GGC TAC CAC CCC TTG  **Reverse:** CCA GCA CCC AAG GTC ACA CCA AAG C |
| **A_2A_R** | NM_009630.3 | **Forward:** GCCAGAGCAAGAGGCAGGTAT  **Reverse:** AAGCACGTTACCCAGGATGG |

**S1 Table. Primers used in the qRT-PCR analysis.** The table indicates the gene, the gene accession number and the primer sequence.
