# Supplementary material for: Changes in adenosine receptors and neurotrophic factors in the SOD1G93A mouse model of amyotrophic lateral sclerosis: Modulation by chronic caffeine
Source: PLoS One. 2022 Dec 14;17(12):e0272104. doi: 10.1371/journal.pone.0272104 (PMC9749988; doi:10.1371/journal.pone.0272104)
Supplement: S1 Appendix — (PDF) [file pone.0272104.s002.pdf]

qRT-PCR – A1R Cortex

	4-6 Weeks	4-6 Weeks	4-6 Weeks	4-6 Weeks	4-6 Weeks	4-6 Weeks	12-14 Weeks	12-14 Weeks	12-14 Weeks	12-14 Weeks	12-14 Weeks	12-14 Weeks
WT	0,31	0,67	1,45	1,34	0,74		0,92	0,41	1,71	0,83	1,01	1,79
SOD1	2,43	2,11	1,27	1,37	1,08	0,97	1,12	0,73	0,6	0,94	0,71	0,64

qRT-PCR – A1R Spinal Cord

	4-6 Weeks	4-6 Weeks	4-6 Weeks	4-6 Weeks	4-6 Weeks	4-6 Weeks	12-14 Weeks	12-14 Weeks	12-14 Weeks	12-14 Weeks	12-14 Weeks	12-14 Weeks
WT	0,67	0,37	0,98	1,16	1,25	1,12	1,43	0,97	1,03	0,91	0,69	1,11
SOD1	0,89	3,96	1,8	1,81	3,63	0,73	0,88334	1,07899	0,64996	0,14552	0,51104	0,46009

qRT-PCR – A2AR Cortex

	4-6 Weeks	4-6 Weeks	4-6 Weeks	4-6 Weeks	4-6 Weeks	4-6 Weeks	4-6 Weeks	12-14 Weeks	12-14 Weeks	12-14 Weeks	12-14 Weeks	12-14 Weeks	12-14 Weeks	12-14 Weeks
WT	0,4	0,39	0,86	0,86	0,51	2,48*		0,12	1,08	2,08	3,4	0,29		
SOD1	1,36	1,21	0,92	1,03	0,49	0,39	0,67	0,15	0,12	0,04	0,16	0,19	0,28	0,5

qRT-PCR – A2AR Spinal Cord

	4-6 Weeks	4-6 Weeks	4-6 Weeks	4-6 Weeks	4-6 Weeks	4-6 Weeks	4-6 Weeks	12-14 Weeks	12-14 Weeks	12-14 Weeks	12-14 Weeks	12-14 Weeks	12-14 Weeks
WT	0,21	0,12	0,32	0,39	0,42	2,27*		0,3	0,31	0,79	1,08	3,57*	
SOD1	0,23	1,22	0,38	0,68	1,07	0,1	0,47	0,4	0,30885	0,19297	0,10164	0,88949	0,11561

A1R Binding – Cortex (4-6 weeks)

	Wt (4-6 weeks)	Wt (4-6 weeks)	Wt (4-6 weeks	Wt (4-6 weeks)	Wt (4-6 weeks)	Wt (4-6 weeks)	Wt (4-6 weeks)	Wt (4-6 weeks)	SOD(4-6 weeks)	SOD(4-6 weeks)	SOD(4-6 weeks)	SOD(4-6 weeks)	SOD(4-6 weeks)	SOD(4-6 weeks)	SOD(4-6 weeks)
0	-2,588624*	1, 823066	1, 823066	1, 785486	-1,011653*	1, 924346966	0*	2, 18289084	0	-0,5064071*	0,5620295	0,8992472	0,295671695	-0,708969935*	-0,606358567*
0,6	12, 23713	19, 14219	18, 23066	14, 84185	16, 29885	13, 6729916	12, 15377031	21, 8289084	9, 985542	13, 97684	35,07064*	21, 35712	2,562488026*	3,342286835*	16, 73549644
1,8	12, 82546	16, 61015	-8,305077*	10, 15495	36, 98154	25, 72548049	25, 21907339	-9,944280493*	17, 66673	5,266634*	39, 34206	46, 31123	23, 06239223	23, 29472643	6,306129093*
3,6	26, 7099	26, 83958	59, 65475	26, 00113	52, 26875	-133,4889106*	54, 59068497	71, 42903915	27, 39623	48, 71636	82, 61834	62, 16047	31, 045528	32, 00492848	58, 33169411
6	28, 59253	51, 95737	85, 5828	235,7957*	-41,9274*	44, 66510588	26, 73829468	102, 4745978	30, 85276	65, 32652	153, 6589	-28,88832*	55, 8819504	11,34351895*	78, 2202551
12	39, 77068	69, 88418	88, 92509	-0,4463714*	-181,8727*	62, 28807283	62, 69319851	106, 4765643	24, 70782	74, 44184	126, 007	-392,8586*	60, 61269753	49, 93173968	89, 1347093
24	95, 89677		99, 8,6348	97, 97852	287,3095*	148, 9849677	-28,25751597*	119, 5739093		-737,2275*	48,78416*	-326,5392*	141, 4296276	-19,95243959*	-882,7368014*
42		123, 2595	154, 9606	1101,756*	931,5077*	128, 6274024	42,74075892*	185, 5457214	46,98326*	121, 8415	-109,7082*	-1293,23*	131, 1796755	65,63035967*	145, 8898711

A1R Binding – Cortex (12-14 weeks)

	Wt(12-14 Weeks)	Wt(12-14 Weeks)	Wt(12-14 Weeks)	Wt(12-14 Weeks)	SOD(12-14 Weeks)	SOD(12-14 Weeks)	SOD(12-14 Weeks)	SOD(12-14 Weeks)	SOD(12-14 Weeks)	SOD(12-14 Weeks)
0	-0,7890997*	0	-2,543499*	-1,933492*	0,8361108	-0,3462669*	-0,1751463*	0	2, 077793	1, 023056
0,6	5, 81961	9, 293105	15, 62435	31, 70926	10, 12623	3, 808936	6, 130119	0,929776*	32,94786*	12, 48128
1,8	16, 66973	13, 5028	18, 89456	16, 62803	13, 93518	12, 63874	9, 457898	4, 338954	45,11779*	16, 57351
3,6	28, 40759	23, 27248	37, 78912	38, 66983	23, 68981	19, 39095	14, 18685	7, 360726	70,34814*	28, 23634
6	31, 26807	26, 21132	25, 07163	53, 36437	32, 88702	25, 19092	23, 20688	7,593171*	73,01959*	49, 92513
12	55, 53289	31, 93015	55, 59361	59, 55154	45, 14998	32, 46252		15, 18634	58, 77187	49, 72051
24	45, 37323	30, 42102		82, 36674	49, 05183	51, 59377	28, 0234	32, 15475	59, 06869	
42	62, 43751	57, 42662		61, 48503	51, 56017	59, 90417	39, 49548	80, 89051	176,0187684*	80, 82141

A1R Binding – Spinal Cord (4-6 weeks)

	Wt (4-6 weeks)	Wt (4-6 weeks)	Wt (4-6 weeks	Wt (4-6 weeks)	Wt (4-6 weeks)	Wt (4-6 weeks)	Wt (4-6 weeks)	Wt (4-6 weeks)	SOD(4-6 weeks)	SOD(4-6 weeks)	SOD(4-6 weeks)	SOD(4-6 weeks)	SOD(4-6 weeks)	SOD(4-6 weeks)	SOD(4-6 weeks)
0	-2,588624*	1, 823066	1, 823066	1, 785486	-1,011653*	1, 924346966	0*	2, 18289084	0	-0,5064071*	0,5620295	0,8992472	0,295671695	-0,708969935*	-0,606358567*
0,6	12, 23713	19, 14219	18, 23066	14, 84185	16, 29885	13, 6729916	12, 15377031	21, 8289084	9, 985542	13, 97684	35,07064*	21, 35712	2,562488026*	3,342286835*	16, 73549644
1,8	12, 82546	16, 61015	-8,305077*	10, 15495	36, 98154	25, 72548049	25, 21907339	-9,944280493*	17, 66673	5,266634*	39, 34206	46, 31123	23, 06239223	23, 29472643	6,306129093*
3,6	26, 7099	26, 83958	59, 65475	26, 00113	52, 26875	-133,4889106*	54, 59068497	71, 42903915	27, 39623	48, 71636	82, 61834	62, 16047	31, 045528	32, 00492848	58, 33169411
6	28, 59253	51, 95737	85, 5828	235,7957*	-41,9274*	44, 66510588	26, 73829468	102, 4745978	30, 85276	65, 32652	153, 6589	-28,88832*	55, 8819504	11,34351895*	78, 2202551
12	39, 77068	69, 88418	88, 92509	-0,4463714*	-181,8727*	62, 28807283	62, 69319851	106, 4765643	24, 70782	74, 44184	126, 007	-392,8586*	60, 61269753	49, 93173968	89, 1347093
24	95, 89677		99, 8,6348	97, 97852	287,3095*	148, 9849677	-28,25751597*	119, 5739093		-737,2275*	48,78416*	-326,5392*	141, 4296276	-19,95243959*	-882,7368014*
42		123, 2595	154, 9606	1101,756*	931,5077*	128, 6274024	42,74075892*	185, 5457214	46,98326*	121, 8415	-109,7082*	-1293,23*	131, 1796755	65,63035967*	145, 8898711

A1R Binding – Spinal Cord (12-14 weeks)

	Wt(12-14 Weeks)	Wt(12-14 Weeks)	Wt(12-14 Weeks)	Wt(12-14 Weeks)	SOD(12-14 Weeks)	SOD(12-14 Weeks)	SOD(12-14 Weeks)	SOD(12-14 Weeks)	SOD(12-14 Weeks)	SOD(12-14 Weeks)
0	-0,7890997*	0	-2,543499*	-1,933492*	0,8361108	-0,3462669*	-0,1751463*	0	2, 077793	1, 023056
0,6	5, 81961	9, 293105	15, 62435	31, 70926	10, 12623	3, 808936	6, 130119	0,929776*	32,94786*	12, 48128
1,8	16, 66973	13, 5028	18, 89456	16, 62803	13, 93518	12, 63874	9, 457898	4, 338954	45,11779*	16, 57351
3,6	28, 40759	23, 27248	37, 78912	38, 66983	23, 68981	19, 39095	14, 18685	7, 360726	70,34814*	28, 23634
6	31, 26807	26, 21132	25, 07163	53, 36437	32, 88702	25, 19092	23, 20688	7,593171*	73,01959*	49, 92513
12	55, 53289	31, 93015	55, 59361	59, 55154	45, 14998	32, 46252		15, 18634	58, 77187	49, 72051
24	45, 37323	30, 42102		82, 36674	49, 05183	51, 59377	28, 0234	32, 15475	59, 06869	
42	62, 43751	57, 42662		61, 48503	51, 56017	59, 90417	39, 49548	80, 89051	176,0187684*	80, 82141

VEGFA – Cortex

	4-6 Weeks	4-6 Weeks	4-6 Weeks	4-6 Weeks	12-14 Weeks	12-14 Weeks	12-14 Weeks	12-14 Weeks	12-14 Weeks	12-14 Weeks
WT	7,711	5,114	6,95	5,782	2,826	1,854	5,662	6,127	2,145	2,537
SOD1	6,925	4,02	4,535	8,458	3,99	1,761	3,148	2,957	6,609	2,556

VEGFA – Spinal Cord

	4-6 Weeks	4-6 Weeks	4-6 Weeks	4-6 Weeks	4-6 Weeks	4-6 Weeks	12-14 Weeks	12-14 Weeks	12-14 Weeks	12-14 Weeks
WT	9,398	7,577	8,307	7,034	25,741*	2,711	4,525	5,035	7	5,283
SOD1	9,651	8,477	9,948	13,868	2,726	2,708	3,741	5,703	6,425	4,939

VEGFB – Cortex

	4-6 Weeks	4-6 Weeks	4-6 Weeks	4-6 Weeks	12-14 Weeks	12-14 Weeks	12-14 Weeks	12-14 Weeks
WT	461,64	315,389	329,999	349,631	499,295	260,012	352,491	364,224
SOD1	427,126	264,094	314,801	560,783	488,381	249,487	355,572	280,109

VEGFB – Spinal Cord

	4-6 Weeks	4-6 Weeks	4-6 Weeks	4-6 Weeks	12-14 Weeks	12-14 Weeks	12-14 Weeks	12-14 Weeks
WT	585,082	364,351	465,736	691,588	599,069	770,231	624,96	
SOD1	1040,729	1229,019	959,257	1522,546	560,412	542,101	506,795	528,87

VEGFR-1 – Cortex

	4-6 Weeks	4-6 Weeks	4-6 Weeks	4-6 Weeks	12-14 Weeks	12-14 Weeks	12-14 Weeks	12-14 Weeks
WT	1534,832	998,485	1186,215	1359,109	2679,927	2960,5	2687,792	2913,586
SOD1	1526,439	938,072	1133,803	2102,042	2315,435	2044,588	2394,248	1877,024

VEGFR-1 – Spinal Cord

	4-6 Weeks	4-6 Weeks	4-6 Weeks	4-6 Weeks	12-14 Weeks	12-14 Weeks	12-14 Weeks	12-14 Weeks
WT	1766,45	1261,432	1102,992	1149,187	2322,289	2404,229	3303,557	2882,521
SOD1	1812,439	1933,984	1687,088	2495,411	1832,291	2275,969	2777,77	2742,329

VEGFR-2 – Cortex

	4-6 Weeks	4-6 Weeks	4-6 Weeks	4-6 Weeks	4-6 Weeks	4-6 Weeks	12-14 Weeks	12-14 Weeks	12-14 Weeks	12-14 Weeks
WT	379,586	445,796	319,822	556,68	258,066	306,822	406,746	439,72	450,929	385,434
SOD1	336,409	425,576	288,902	302,364	276,122	327,395	369,711	385,904	353,645	338,234

VEGFR-2 – Spinal Cord

	4-6 Weeks	4-6 Weeks	4-6 Weeks	4-6 Weeks	12-14 Weeks	12-14 Weeks	12-14 Weeks	12-14 Weeks
WT	518,709	510,699	432,407	456,874	326,202	318,162	527,072	323,862
SOD1	539,445	740,586	894,286	848,514	323,359	370,66	423,855	417,695

VEGFA caffeine– Cortex

	vehicle	vehicle	vehicle	vehicle	caffeine	caffeine	caffeine	caffeine	caffeine
WT	3,22156937	4,10485918	4,44940412	2,68512983	1,0894167	1,65058065	2,28589663	2,19440777	3,01666175
SOD	1,72220387	0,6608608	1,69896755	1,88710592	1,73659163	2,74395064	2,24073938	1,90742956	1,72059495

VEGFA caffeine– Spinal Cord

	vehicle	vehicle	vehicle	vehicle	vehicle	caffeine	caffeine	caffeine	caffeine	caffeine
WT	2,22345433	2,45846927	2,56253011	3,91658615	2,82644589	5,22874876	4,23492276	2,56479684	3,301846982	3,54006253
SOD	6,28566511	2,34633524	3,66939801	4,30163735		2,47290166	7,74432142	2,19385539	3,224541315	3,078424466

VEGFB caffeine– Cortex

	vehicle	vehicle	vehicle	vehicle	vehicle	caffeine	caffeine	caffeine	caffeine	caffeine
WT	166,980744	209,233792	127,336662	113,210614	216,988192	151,277572	156,44214	117,596761	128,414023	299,854852
SOD	174,716561	132,535437	172,49807	178,916727		146,979124	183,510216	107,963915	172,116454	178,300801

VEGFB caffeine– Spinal Cord

	vehicle	vehicle	vehicle	vehicle	vehicle	caffeine	caffeine	caffeine	caffeine	caffeine
WT	280,174118	220,755635	527,895128	516,009914	285,369628	635,510742	185,067917	299,363773	189,612439	522,802342
SOD	591,787371	498,275752	473,976625	618,460145		454,317044	688,571963	402,167255	524,848508	656,538669

VEGFR-1 caffeine– Cortex

	vehicle	vehicle	vehicle	vehicle	vehicle	caffeine	caffeine	caffeine	caffeine	caffeine
WT	1219,82593	1214,21157	1009,24385	750,78334	1198,03397	949,106744	910,381805	800,126346	782,7261	1578,0244
SOD	889,923052	847,180613	991,6976	891,505129		769,033902	1071,94933	657,386357	1110,8675	1023,13303

VEGFR-1 caffeine– Spinal Cord

	vehicle	vehicle	vehicle	vehicle	vehicle	caffeine	caffeine	caffeine	caffeine	caffeine
WT	628,61152	476,877437	800,686208	874,054204	759,433024	1283,76906	818,79624	943,684389	606,091821	792,548342
SOD	1037,30122	847,044879	824,979958	967,911815		936,428878	1082,93282	730,047965	1436,12667	1053,5979

VEGFR-2 caffeine– Cortex

	vehicle	vehicle	vehicle	vehicle	vehicle	caffeine	caffeine	caffeine	caffeine	caffeine
WT	46,9570792	54,0188015	33,2053291	38,7809991	52,7606541	54,2614734	33,8558774	36,4750688	30,8588054	39,6583478
SOD	50,9580737	34,8321618	59,4943186	36,347376		51,4651434	73,9722941	35,1944343	54,0711063	37,8471284

VEGFR-2 caffeine– Spinal Cord

	vehicle	vehicle	vehicle	vehicle	vehicle	caffeine	caffeine	caffeine	caffeine	caffeine
WT	64,7203308	50,1479995	207,108836	164,096766	54,3742293	251,207276	52,4582016	75,1013062	45,2620515	179,389486
SOD	133,378554	89,4888381	108,154913	139,183133		159,976168	153,829903	98,9144887	170,288419	81,7891368

BDNF caffeine– Cortex

	vehicle	vehicle	vehicle	vehicle	vehicle	caffeine	caffeine	caffeine	caffeine	caffeine	caffeine
WT	25,3585621	22,3522386	17,8215741	21,165478	18,1012942	19,0705617	33,5120584	43,8651172	53,5815196	51,191361	
SOD	16,5961503	37,7283389	21,1171093	34,4627465	38,8277494	7,69722449	14,7981076	18,2740037	20,6128732	6,01282445	15,6805602

BDNF caffeine– Spinal Cord

	vehicle	vehicle	vehicle	vehicle	vehicle	caffeine	caffeine	caffeine	caffeine	caffeine	caffeine	caffeine
WT	5,78763291	12,682925	14,7470243	13,5358396	9,14588166	12,7706753	15,7938237	34,7755339	7,84143461	12,3243369	2,68558978	
SOD	17,9642715	17,3919648	20,2883456	33,9486871	7,09322103	82,8370994	61,5821945	89,6148842	22,9961827	30,2532173	34,3813157	22,0433654
