# Supplementary material for: Changes in adenosine receptors and neurotrophic factors in the SOD1G93A mouse model of amyotrophic lateral sclerosis: Modulation by chronic caffeine
Source: PLoS One. 2022 Dec 14;17(12):e0272104. doi: 10.1371/journal.pone.0272104 (PMC9749988; doi:10.1371/journal.pone.0272104)

A$_{2A}$R gel 1 and 2

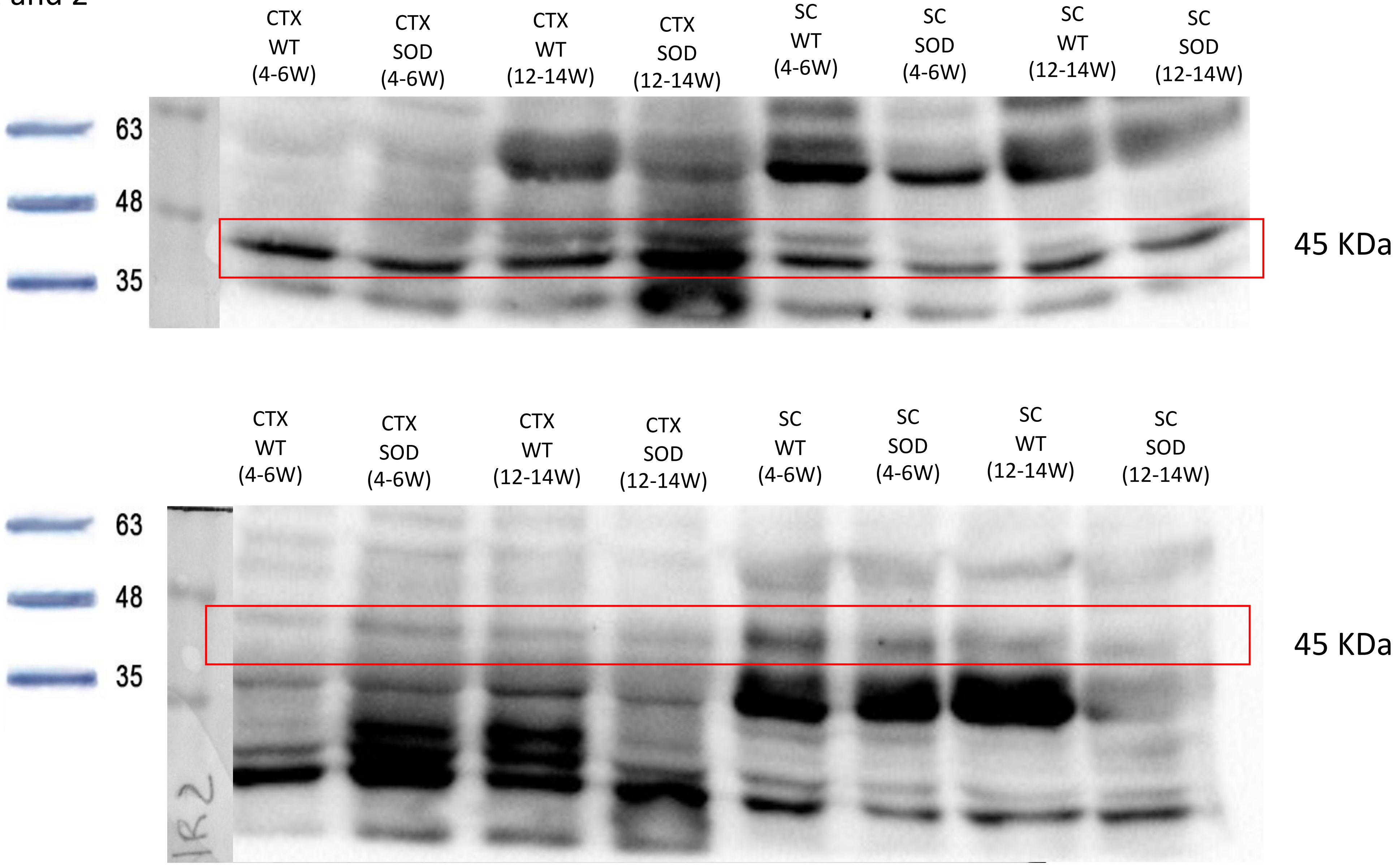

Densitometry values $A_{2A}R$/GAPDH gel 1

		A2AR	GAPDH	A2AR/GAPDH		A2AR
	WT(4-6w)	83,135	144,054	0,57710997		1
	Pre-S	169,025	166,451	1,01546401		1,759567602
	WT(12-14w)	143,041	161,959	0,88319266		1
	S	105,569	136,934	0,77094805		0,872910389
	CTX					
	WT(4-6w)	62,802	133,946	0,46886059		1
	Pre-S	37,658	190,503	0,19767668		0,42161078
	WT(12-14w)	34,824	167,943	0,20735607	FOLD	1
	S	44,689	101,795	0,43900977		2,117178342
Gel 1	SC					

Densitometry values $A_{2A}R$/GAPDH gel 2

		A2AR	GAPDH	A2AR/GAPDH		A2AR
	WT(4-6w)	83,243	138,778	0,599828503		1
	Pre-S	169,436	116,431	1,455248173		2,426107
	WT(12-14w)	145,098	205,49	0,706107353		1
	S	107,485	160,494	0,66971351		0,948458
	CTX					
	WT(4-6w)	61,011	125,503	0,486131806		1
	Pre-S	36,97	88,897	0,415874551		0,855477
	WT(12-14w)	31,778	78,7	0,403786531	FOLD	1
	S	37,8	77,39	0,488435198		1,209637
Gel 2	SC					

GAPDH gel 1 and 2

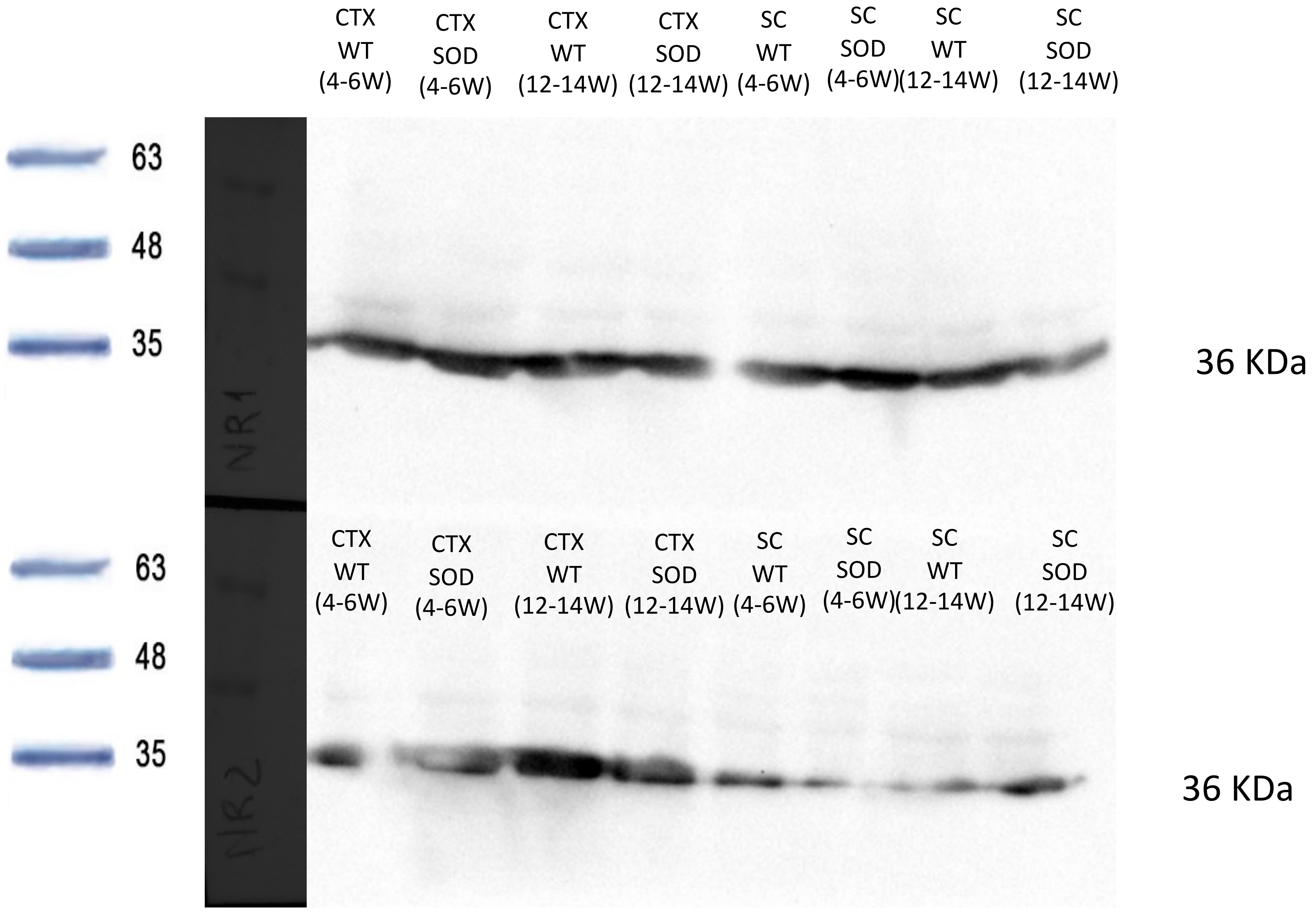

$A_{2A}R$ gel 3 and 4

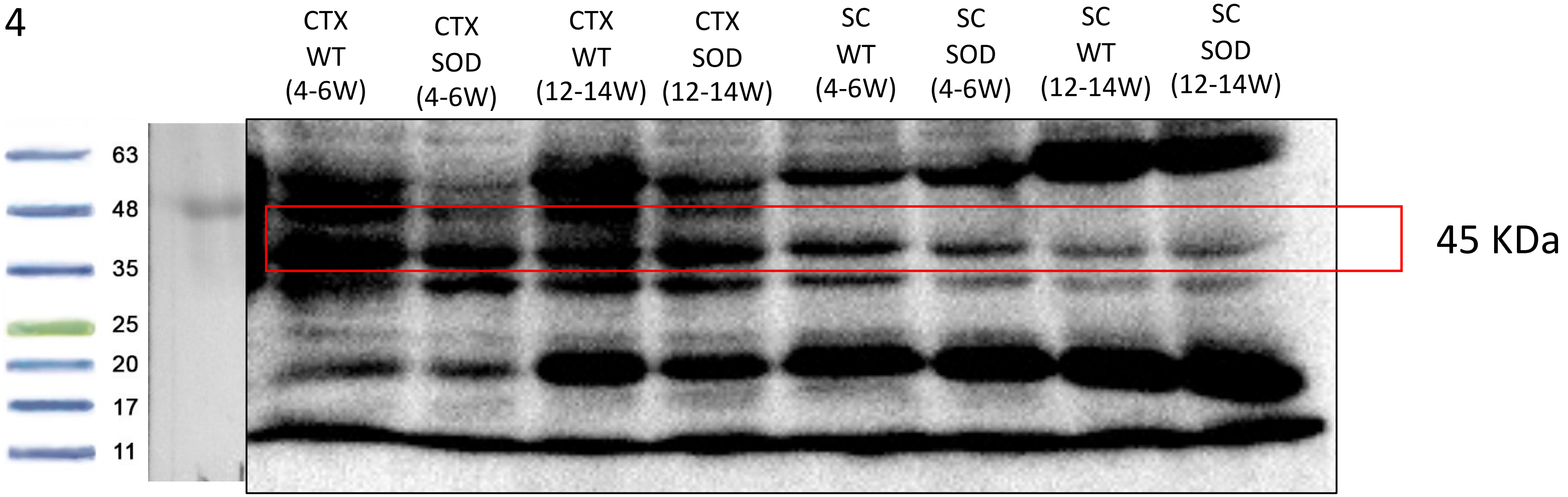

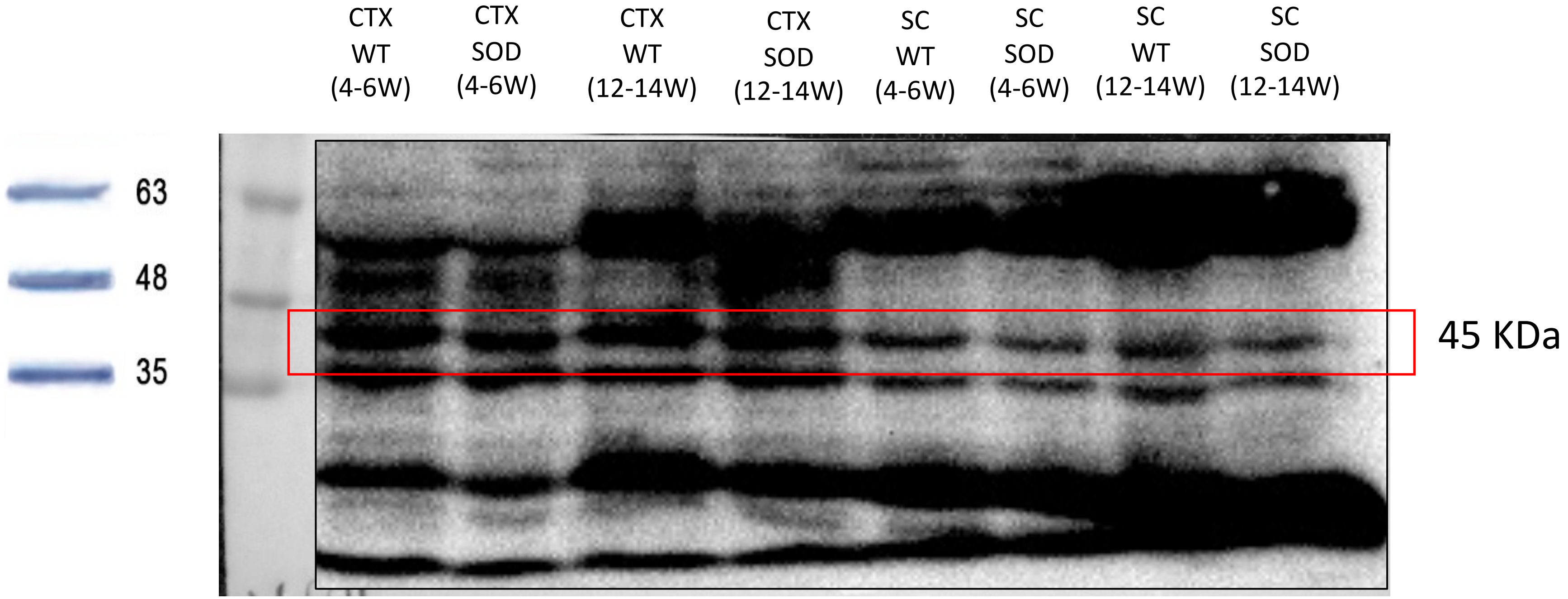

Densitometry values $A_{2A}R$/GAPDH gel 3

		A2AR	GAPDH	A2AR/GAPDH		A2AR
	WT(4-6w)	40,09	215,056	0,186416561		1
	Pre-S	36,344	156,25	0,2326016		1,247752
	WT(12-14w)	30,289	125,222	0,241882417		1
	S	33,148	137,243	0,241527801		0,998534
	CTX					
	WT(4-6w)	24,263	110,378	0,219817355		1
	Pre-S	17,332	127,272	0,136180778		0,619518
	WT(12-14w)	12,893	130,88	0,098510086	FOLD	1
	S	13,656	134,425	0,101588246		1,031247
Gel 3	SC					

Densitometry values $A_{2A}R$/GAPDH gel 4

		A2AR	GAPDH	A2AR/GAPDH		A2AR
	WT(4-6w)	26,34	212,029	0,12422829		1
	Pre-S	24,677	149,605	0,164947696		1,327779
	WT(12-14w)	48,696	163,428	0,297966077		1
	S	42,313	174,73	0,242162193		0,812717
	CTX					
	WT(4-6w)	69,904	177,38	0,39409178		1
	Pre-S	100,68	189,688	0,530766311		1,346809
	WT(12-14w)	165,324	169,431	0,975760044	FOLD	1
	S	168,558	147,996	1,138936187		1,16723
Gel 4	SC					

GAPDH gel 3 and 4

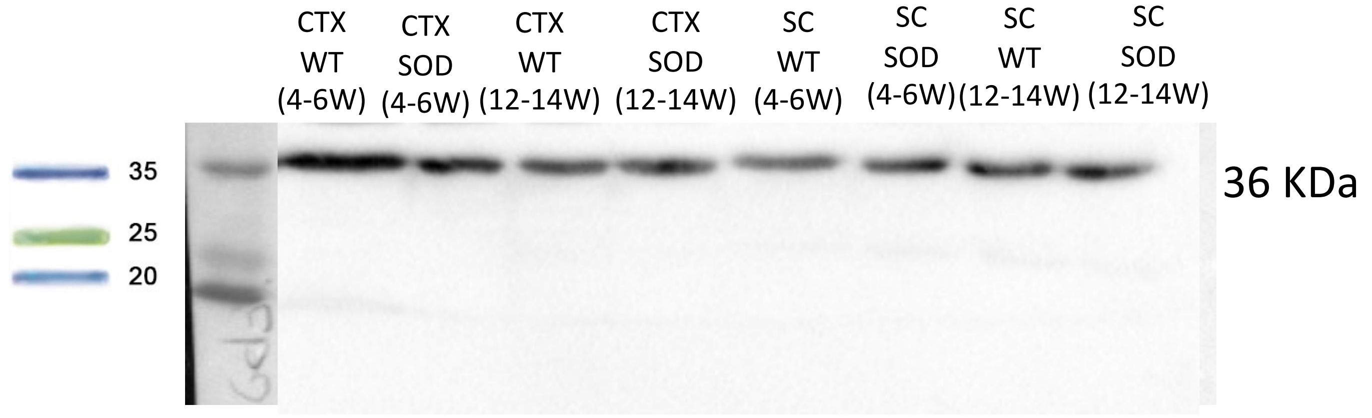

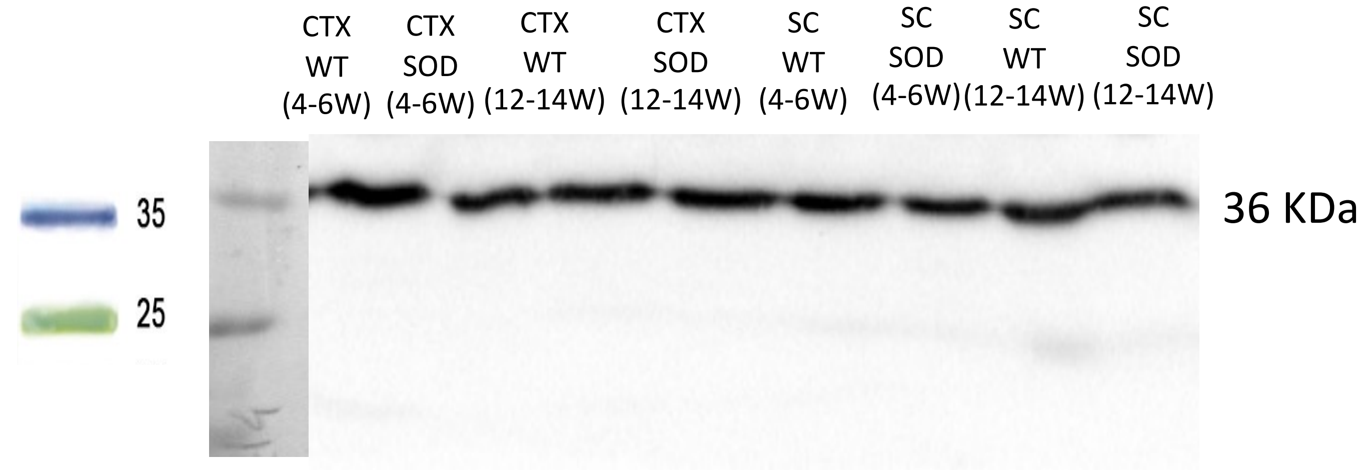

Gel 4 – Spinal Cord (SC)_Figure 2F

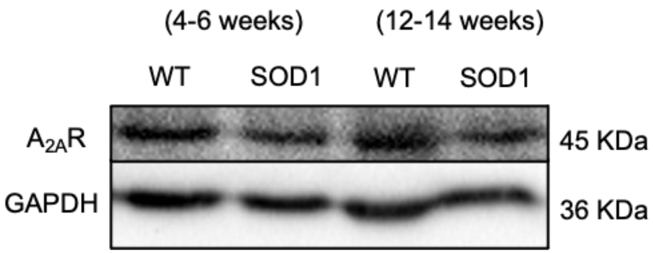

$A_{2A}R$ gel 5

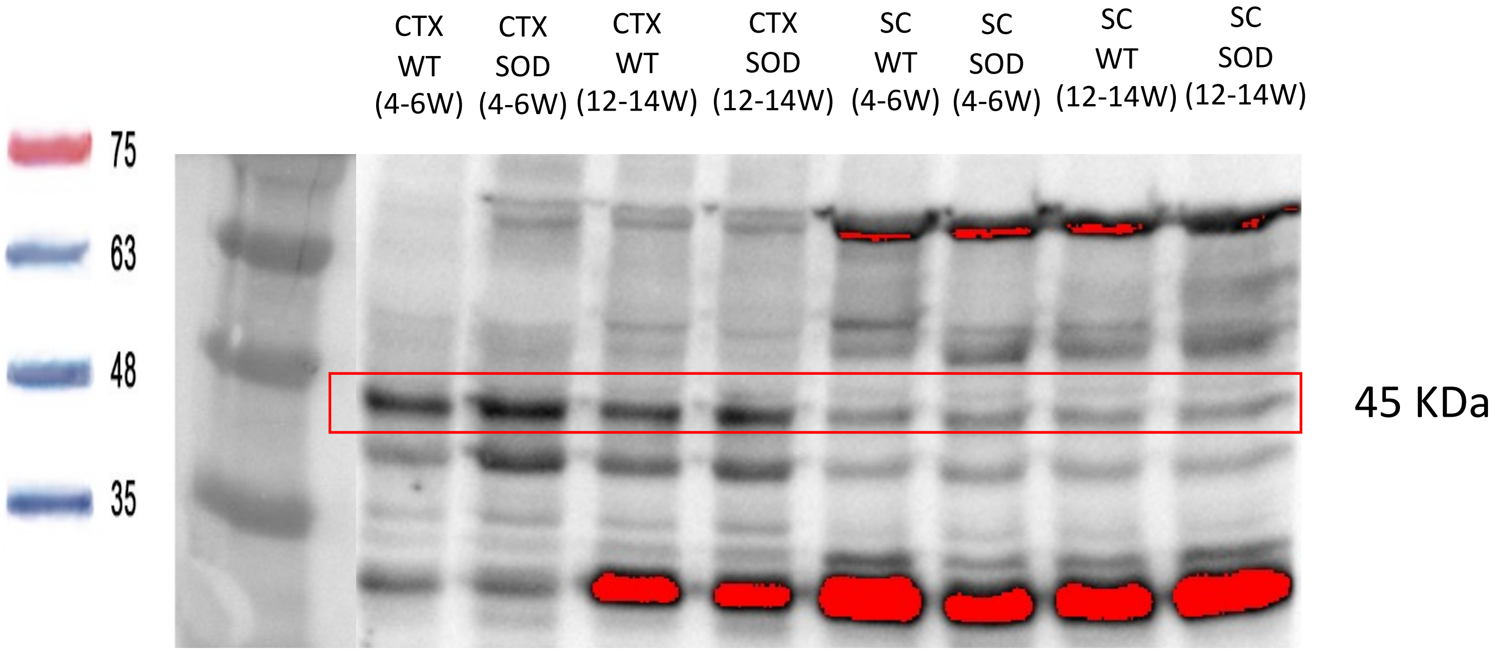
CTX WT (4-6W)
CTX SOD (4-6W)
CTX WT (12-14W)
CTX SOD (12-14W)
SC WT (4-6W)
SC SOD (4-6W)
SC WT (12-14W)
SC SOD (12-14W)
75
63
48
35
45 KDa

GAPDH gel 5

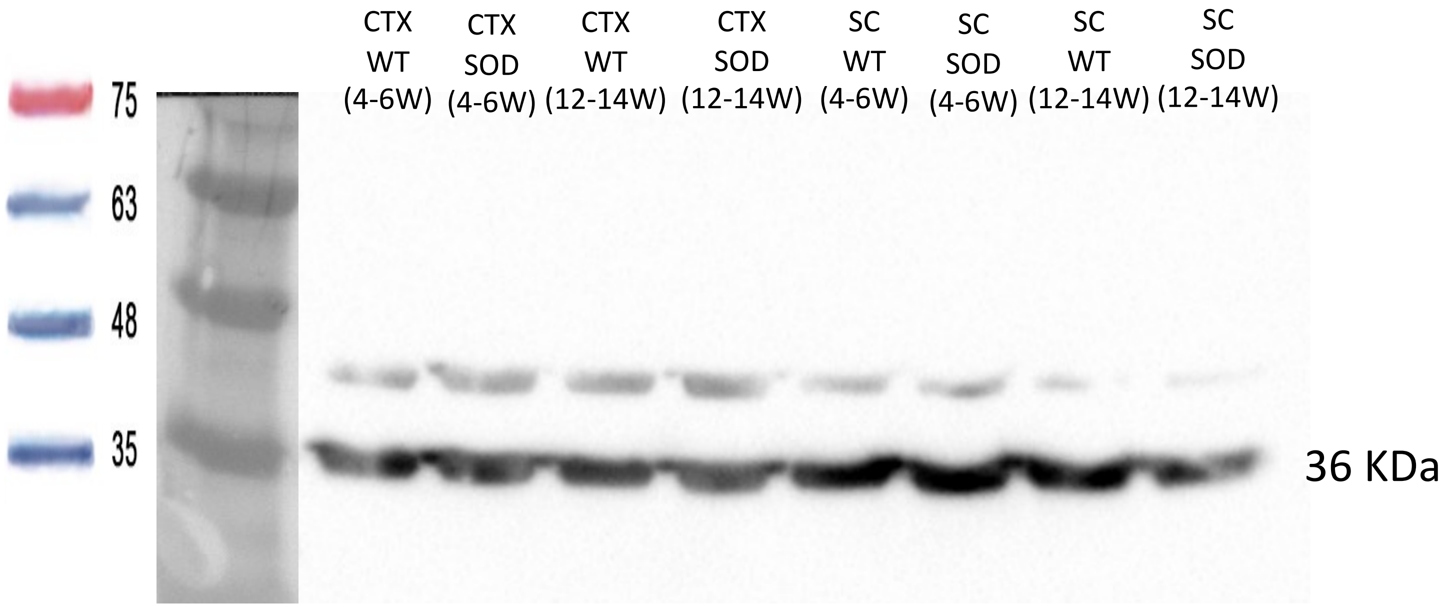

CTX WT (4-6W)
CTX SOD (4-6W)
CTX WT (12-14W)
CTX SOD (12-14W)
SC WT (4-6W)
SC SOD (4-6W)
SC WT (12-14W)
SC SOD (12-14W)
75
63
48
35
36 KDa

Densitometry values $A_{2A}R$/GAPDH gel 5

		A2AR	GAPDH	A2AR/GAPDH		A2AR
	WT(4-6w)	132,27	132,284	0,999894167		1
	Pre-S	155,602	148,282	1,049365398		1,049476
	WT(12-14w)	111,113	141,007	0,787996341		1
	S	120,319	146,959	0,81872495		1,038996
	CTX					
	WT(4-6w)	73,93	184,31	0,401117682		1
	Pre-S	76,689	201,543	0,38050937		0,948623
	WT(12-14w)	68,048	191,578	0,355197361	FOLD	1
	S	72,786	116,961	0,622310001		1,752012
Gel 5	SC					

Gel 5 – Cortex (CTX)_Figure 1F

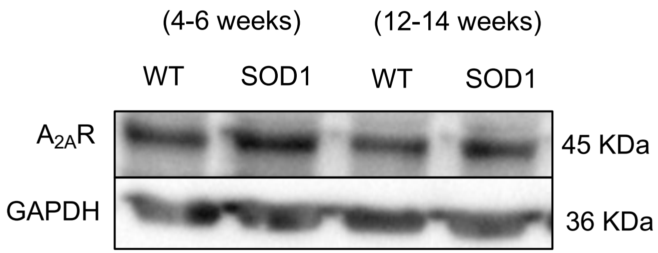

A_1R CTX caffeine gel 1 and 2

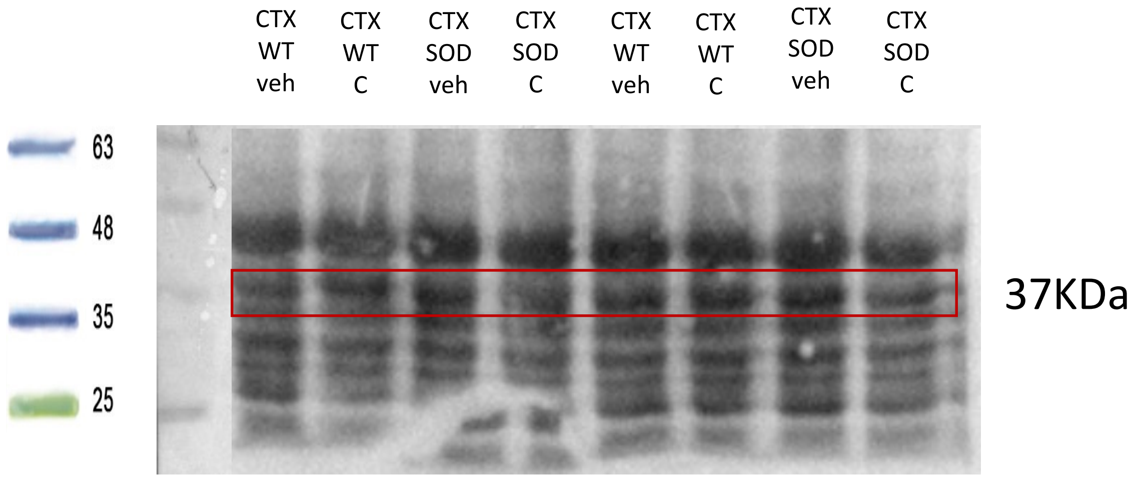

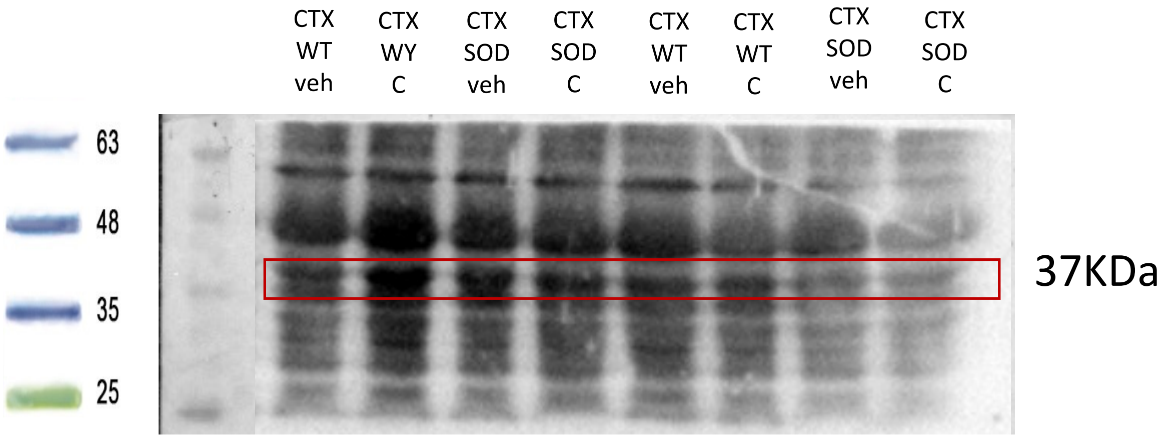

GAPDH CTX caffeine gel 1 and 2

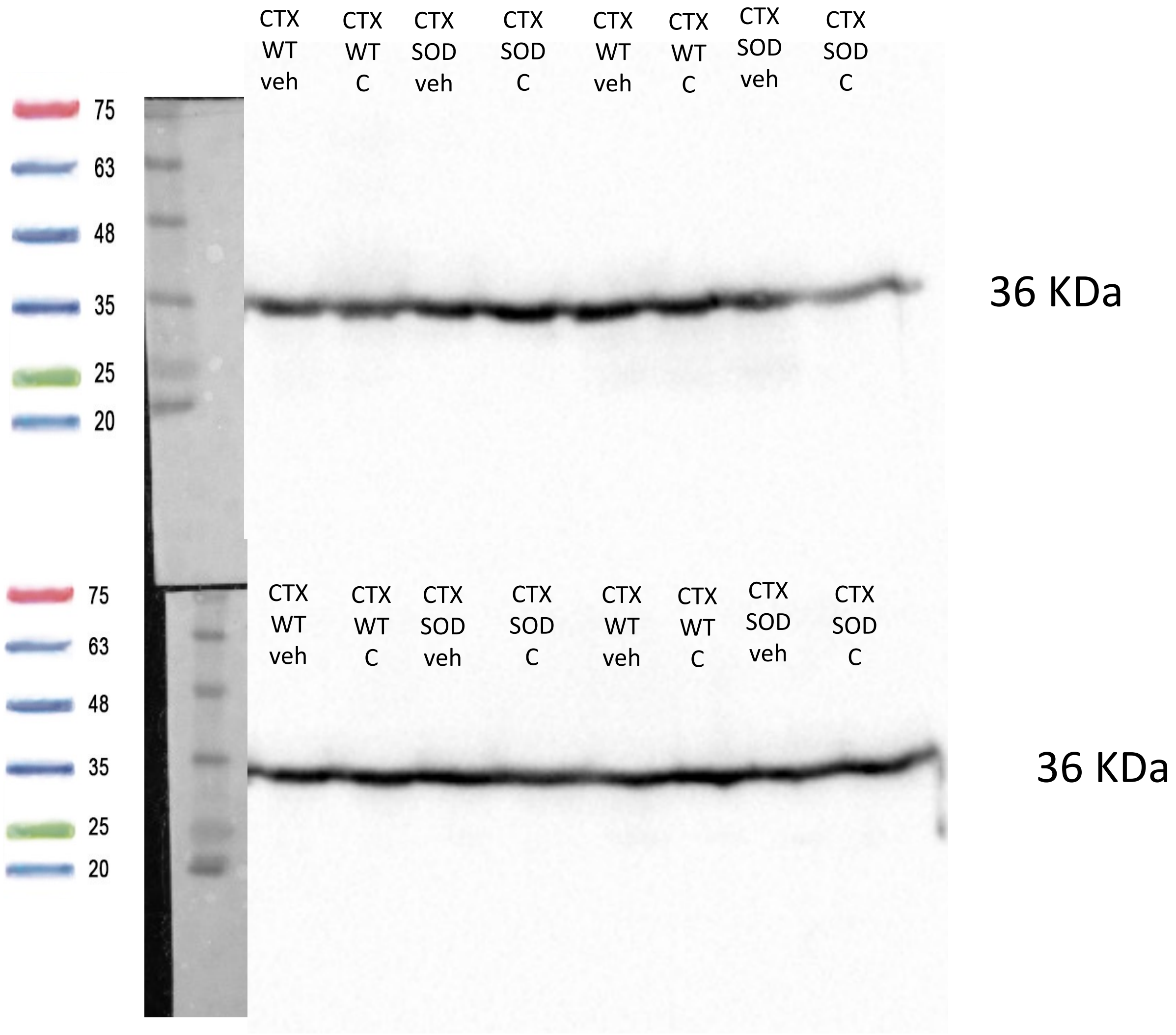

GAPDH is the same for A_1R and $A_{2A}R$ caffeine (stripping Western blot membranes)

Densitometry values A_1R/GAPDH gel 1

		A1R	GAPDH	A1R/GAPDH		A1R
	WT1 NC	175,447	125,935	1,393155199		1
	WT1 C	208,634	131,87	1,582118753		1,135637
	SOD 1 NC	167,332	168,471	0,993239193		0,712942
	SOD 1 C	189,898	188,61	1,006828906		0,722697
	CTX					
	WT 2 NC	188,886	182,395	1,035587598		1
	WT 2 C	195,481	177,215	1,103072539		1,065166
	SOD 2 NC	137,1	172,215	0,796097901	FOLD	0,76874
	SOD 2 C	119,735	106,457	1,124726415		1,086076
Gel 1	CTX					

Densitometry values A_1R/GAPDH gel 2

		A1R	GAPDH	A1R/GAPDH		A1R
	WT 3 NC	207,356	171,457	1,209376112		1
	WT 3 C	242,476	133,989	1,809670943		1,496367
	SOD 3 NC	189,632	213,114	0,889814841		0,735764
	SOD 3 C	224,584	181	1,24079558		1,02598
	CTX					
	WT 4 NC	221,787	198,754	1,115886976		1
	WT 4 C	245,276	173,534	1,413417544		1,266631
	SOD 4 NC	128,775	192,693	0,668291012	FOLD	0,598888
	SOD 4 C	128,836	147,258	0,874899836		0,78404
Gel 2	CTX					

Gel 2 (1st part of the gel) – Cortex (CTX)_Figure 7A

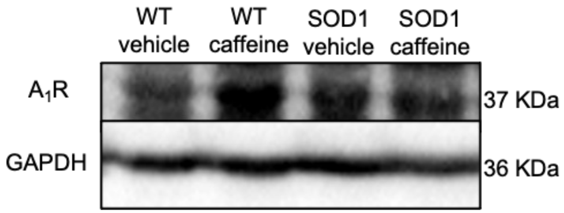

A_1R SC caffeine gel 3 and 4

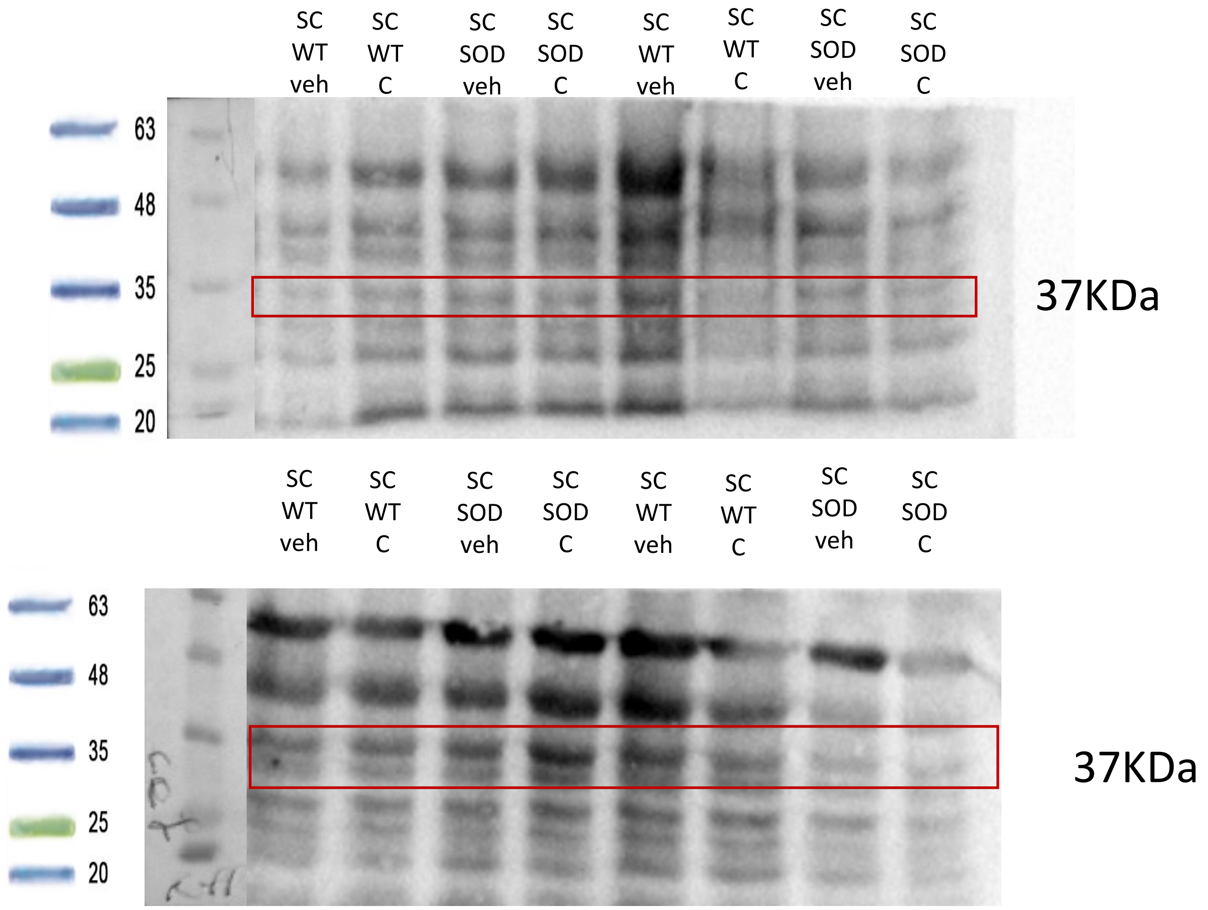

GAPDH SC caffeine gel 3 and 4

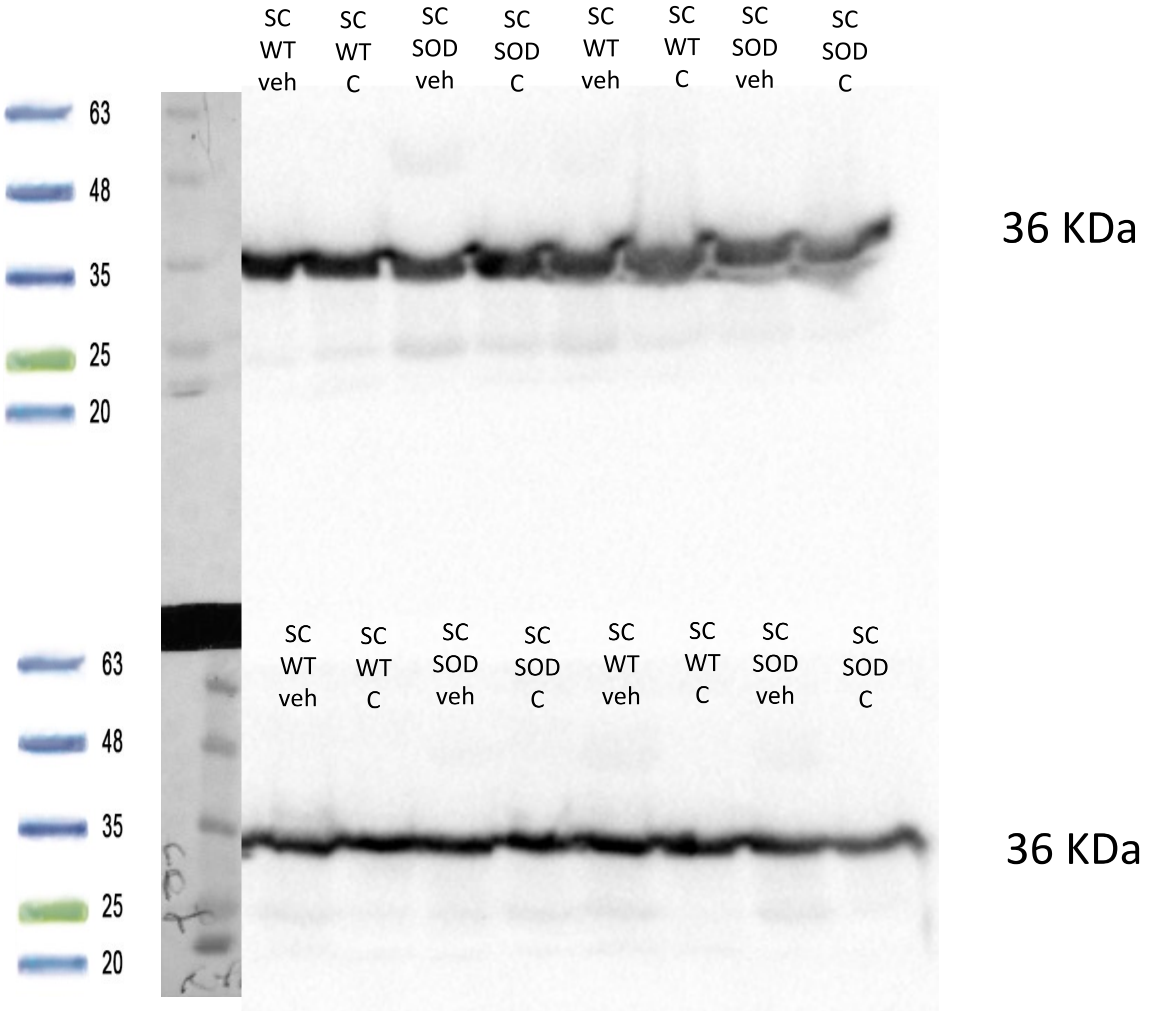

GAPDH is the same for A1R and $A_{2A}R$ caffeine (stripping Western blot membranes)

Densitometry values A_1R/GAPDH gel 3

		A1R	GAPDH	A1R/GAPDH		A1R
	WT 1 NC	90,912	203,124	0,44756897		1
	WT 1 C	153,197	195,097	0,78523504		1,754445
	SOD 1 NC	122,514	166,507	0,73578889		1,643968
	SOD 1 C	121,806	197,517	0,61668616		1,377857
	SC					
	WT 2 NC	173,95	175,86	0,98913909		1
	WT 2 C	109,928	128,532	0,85525783		0,864649
	SOD 2 NC	119,297	138,254	0,86288281		0,872357
	SOD 2 C	121,374	99,664	1,21783192	FOLD	1,231204
Gel 3	SC					

Densitometry values A_1R/GAPDH gel 4

		A1R	GAPDH	A1R/GAPDH		A1R
	WT 3 NC	221,767	189,7	1,16904059		1
	WT 3 C	207,574	210,261	0,987220645		0,8444708
	SOD 3 NC	179,796	152,937	1,175621334		1,00562918
	SOD 3 C	228,736	207,642	1,101588311		0,94230117
	SC					
	WT 4 NC	234,171	196,25	1,193228025		1
	WT 4 C	223,697	196,445	1,138725852		0,95432376
	SOD 4 NC	123,398	168,424	0,732662803	FOLD	0,61401743
	SOD 4 C	86,404	108,836	0,793891727		0,66533111
Gel 4	SC					

Gel 3(1st part of the gel) – Spinal Cord (SC)_Figure 7B

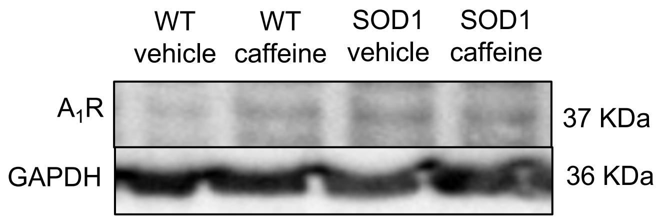

A_1R CTX+SC caffeine gel 5

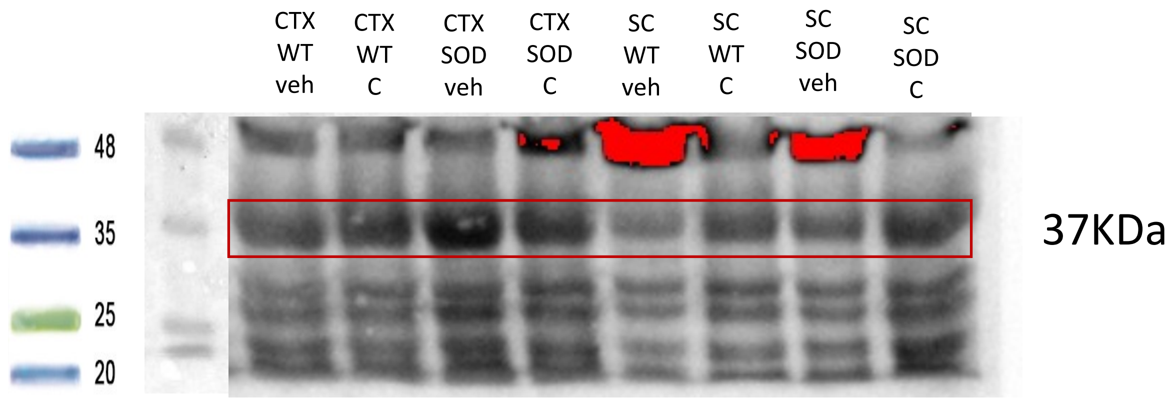

GAPDH CTX + SC caffeine gel 5

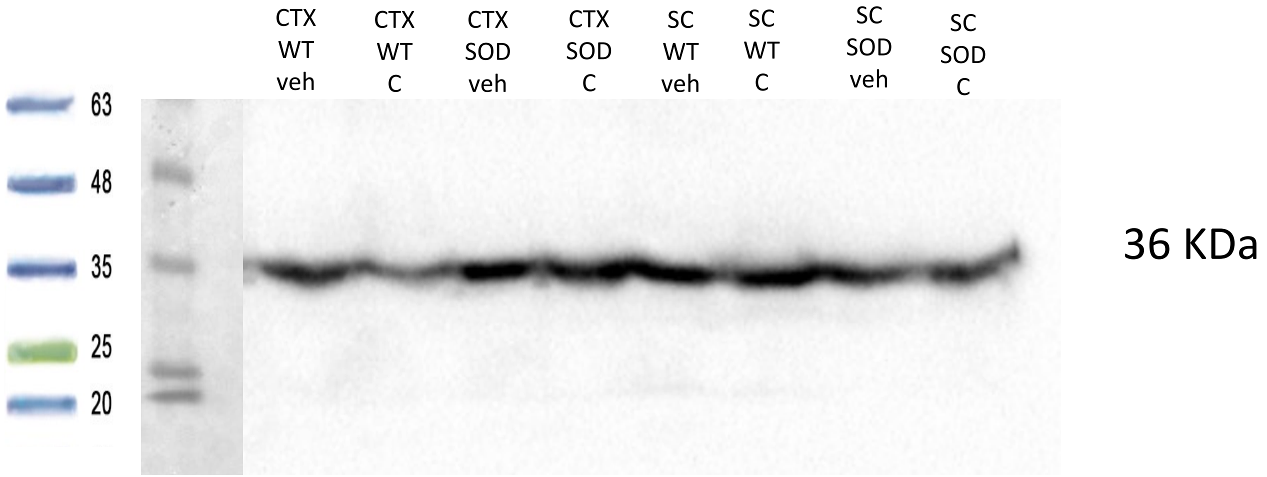

GAPDH is the same for A_1R and $A_{2A}R$ caffeine (stripping Western blot membranes)

Densitometry values A_1R/GAPDH gel 5

		A1R	GAPDH	A1R/GAPDH		A1R
	WT 5 NC	133,495	152,54	0,875147502		1
	WT 5 C	150,374	117,972	1,274658394		1,456507
	SOD 5 NC	137,656	234,478	0,587074267		0,670829
	SOD 5 C	216,105	182,641	1,183222825		1,352027
	CTX					
	WT 5 NC	157,103	179,415	0,875640275		1
	WT 5 C	195,703	187,453	1,044011032		1,192283
	SOD 5 NC	168,103	159,312	1,055181028	FOLD	1,205039
	SOD 5 C	195,721	155,368	1,259725297		1,438633
Gel 5	SC					

$A_{2A}R$ CTX caffeine gel 1 and 2

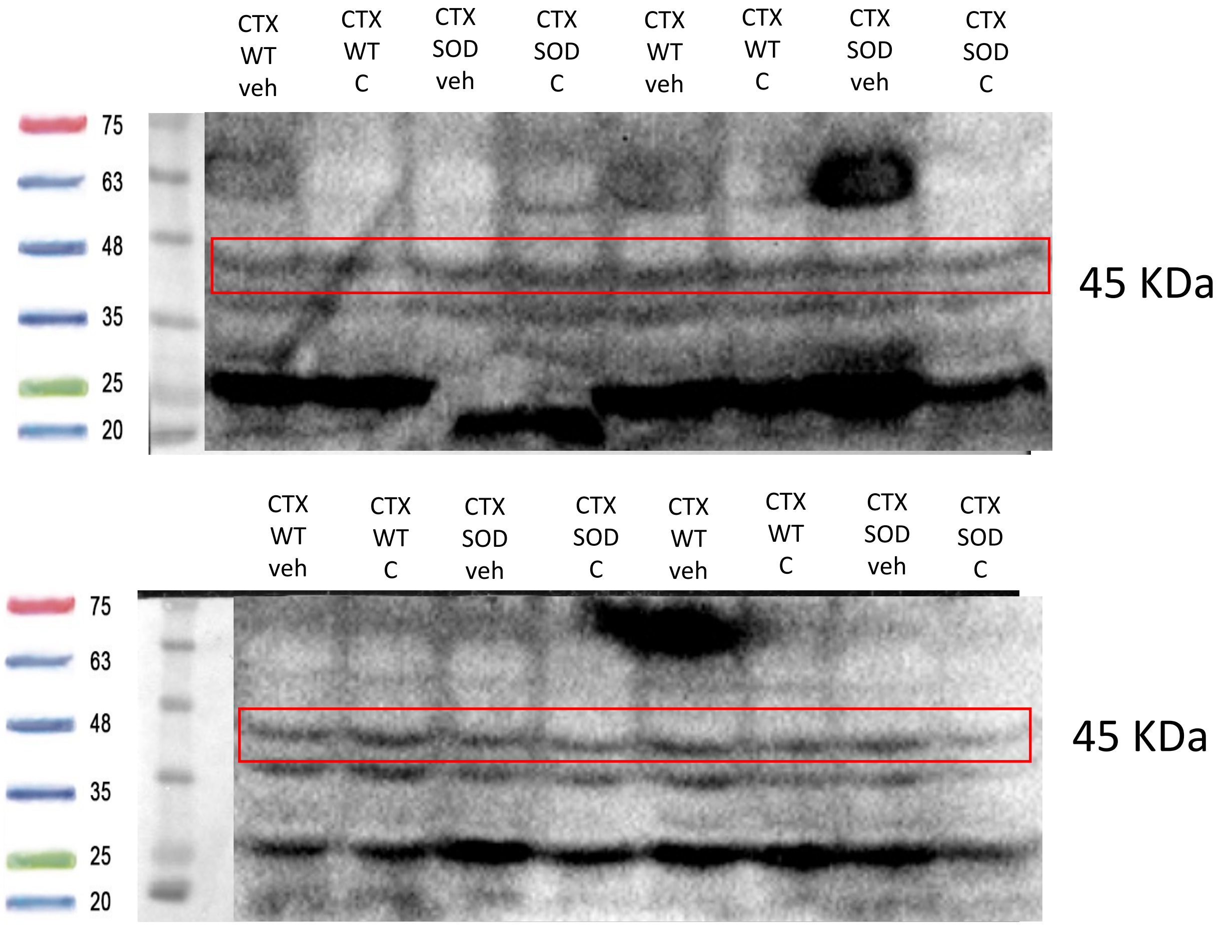

GAPDH CTX caffeine gel 1 and 2

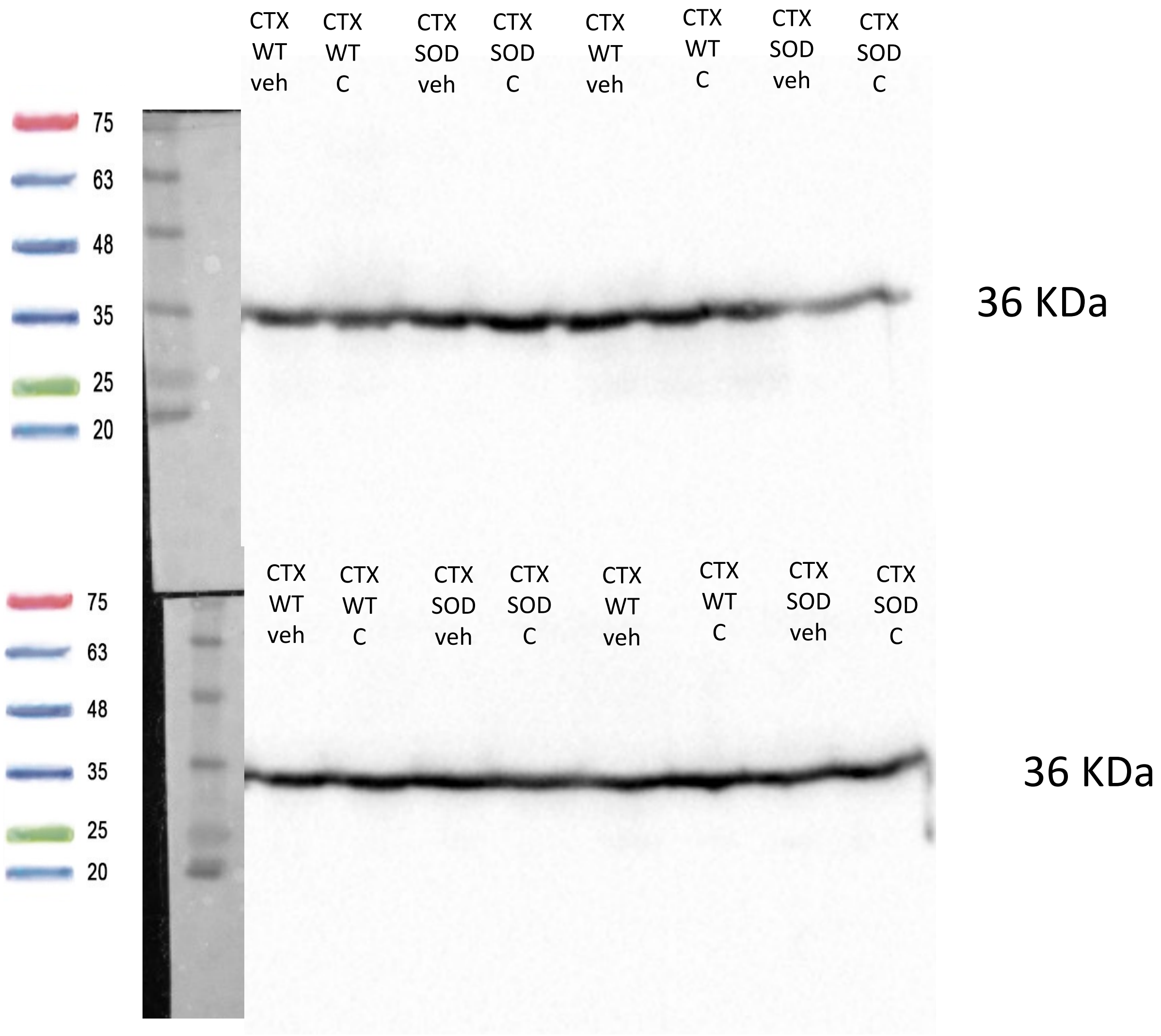

GAPDH is the same for A_1R and $A_{2A}R$ caffeine (stripping Western blot membranes)

Densitometry values $A_{2A}R$/GAPDH gel 1

		A2AR	GAPDH	A2AR/GAPDH		A2AR
	WT1 NC	90,967	125,935	0,72233295		1
	WT1 C	97,112	131,87	0,736422234		1,019505
	SOD 1 NC	73,953	168,471	0,438965757		0,607706
	SOD 1 C	96,019	188,61	0,509087535		0,704782
	CTX					
	WT 2 NC	86,281	182,395	0,473044765		1
	WT 2 C	86,281	177,215	0,486871879		1,02923
	SOD 2 NC	78,569	136,593	0,575205172		1,215964
	SOD 2 C	61,053	77,012	0,792772555	FOLD	1,675893
Gel 1	CTX					

Densitometry values $A_{2A}R$/GAPDH gel 2

		A2AR	GAPDH	A2AR/GAPDH		A2AR
	WT 3 NC	80,393	171,457	0,46888141		1
	WT 3 C	99,682	207,121	0,481274231		1,026431
	SOD 3 NC	93,32	213,114	0,437887703		0,933899
	SOD 3 C	82,573	181	0,45620442		0,972963
	CTX					
	WT 4 NC	114,747	198,754	0,577331777		1
	WT 4 C	102,065	217,146	0,470029381		0,814141
	SOD 4 NC	106,195	192,693	0,551109796		0,954581
	SOD 4 C	68,955	147,258	0,468259789	FOLD	0,811076
Gel 2	CTX					

Gel 2 (1st part of the gel) – Cortex (CTX)_Figure 7C

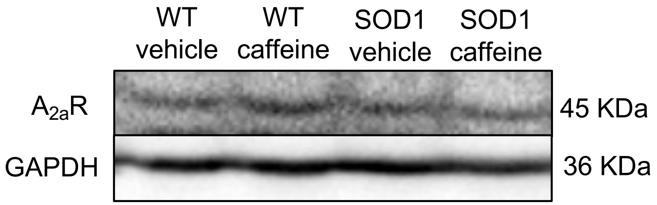

A$_{2A}$R SC caffeine gel 3 and 4

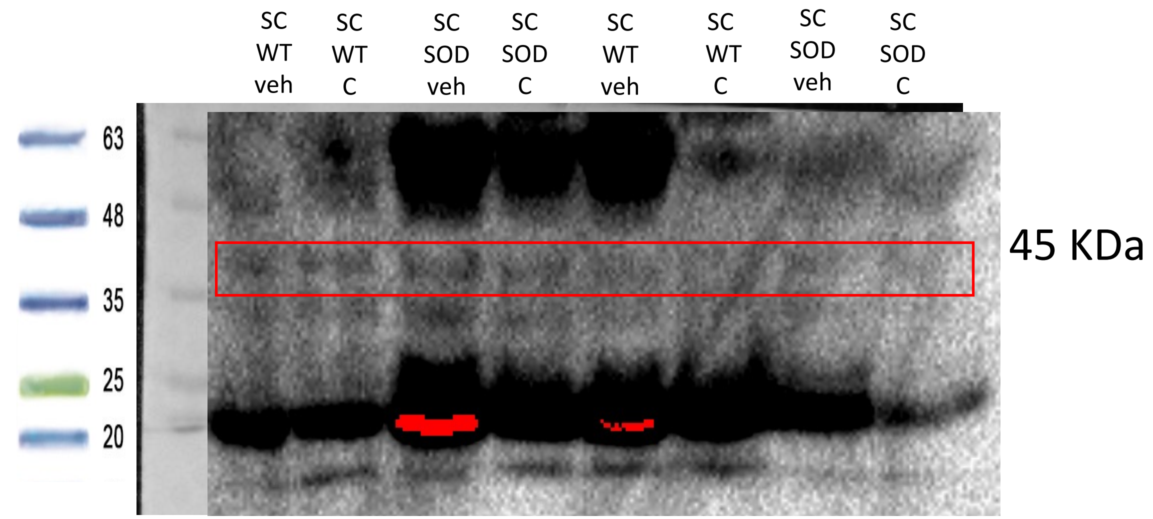

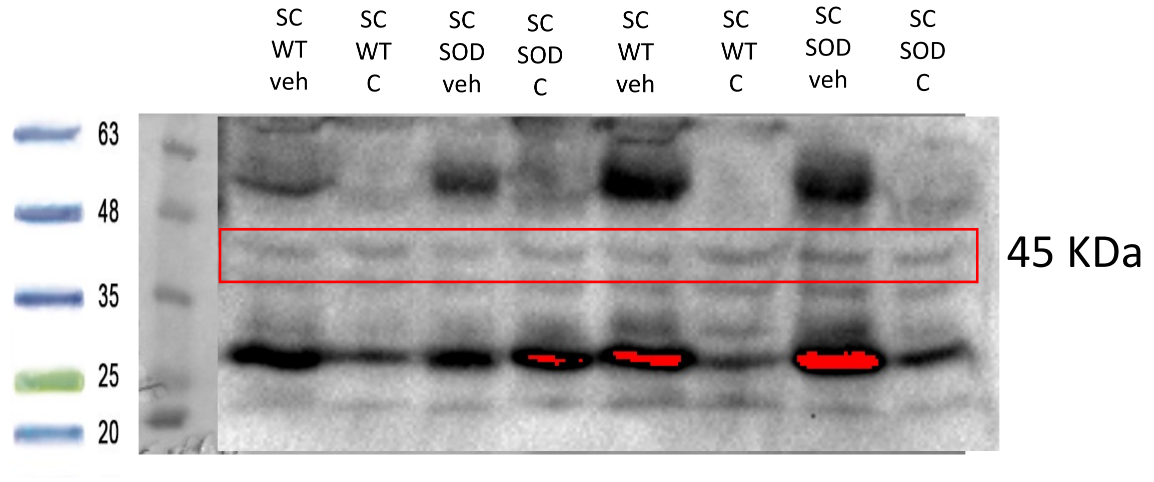

GAPDH SC caffeine gel 3 and 4

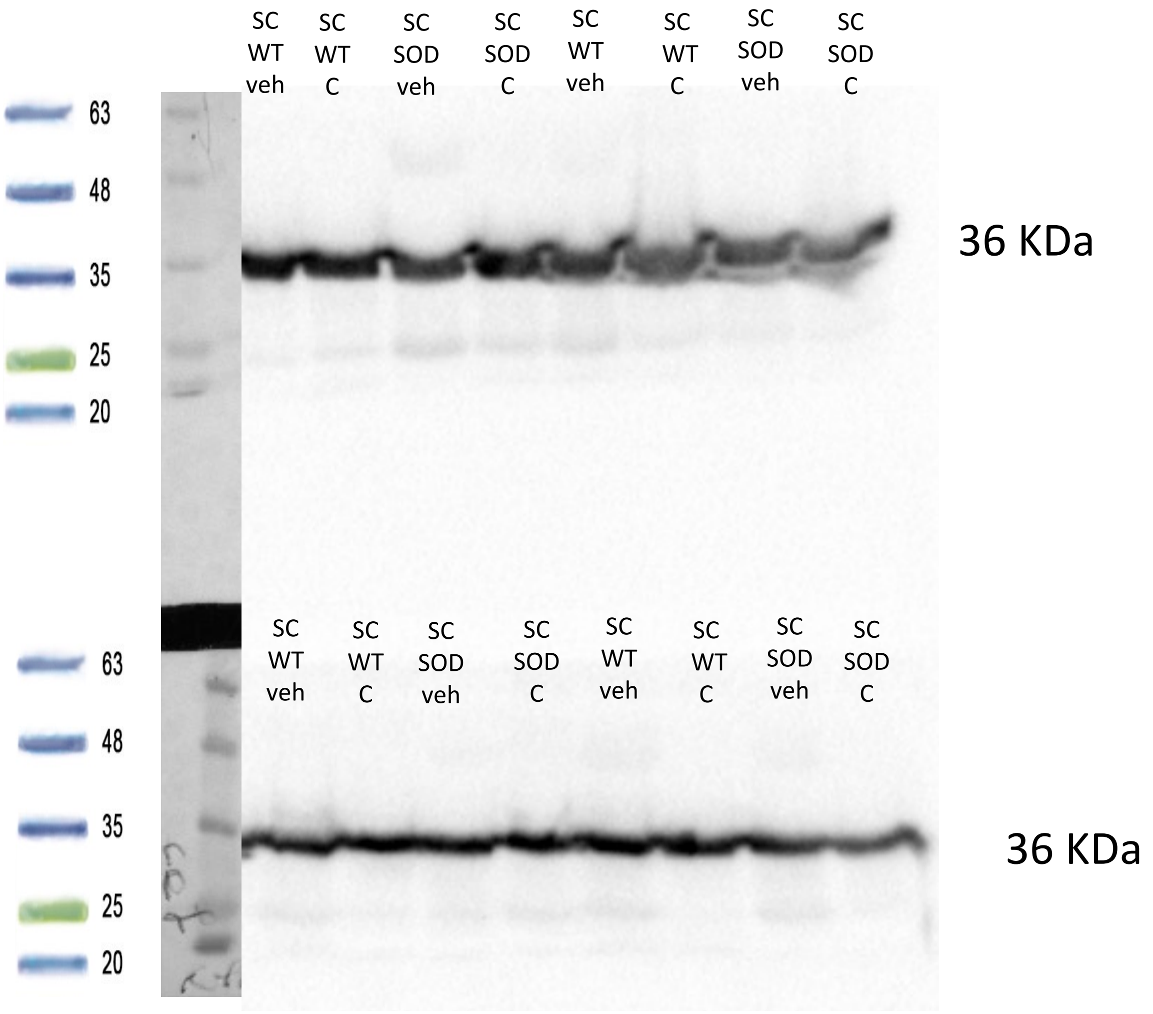

GAPDH is the same for A_1R and $A_{2A}R$ caffeine (stripping Western blot membranes)

Densitometry values $A_{2A}R$/GAPDH gel 3

		A2AR	GAPDH	A2AR/GAPDH		A2AR
	WT 1 NC	67,28	203,124	0,331226246		1
	WT 1 C	71,839	195,097	0,368221961		1,111693
	SOD 1 NC	102,906	166,507	0,618028071		1,865879
	SOD 1 C	107,765	197,517	0,545598607		1,647208
	SC					
	WT 2 NC	107,407	175,86	0,610752872		1
	WT 2 C	82,552	151,187	0,546025783		0,894021
	SOD 2 NC	97	138,254	0,701607187	FOLD	1,148758
	SOD 2 C	72,469	99,664	0,727133167		1,190552
Gel 3	SC					

Densitometry values $A_{2A}R$/GAPDH gel 4

		A2AR	GAPDH	A2AR/GAPDH		A2AR
	WT 3 NC	48,049	189,7	0,2532894		1
	WT 3 C	45,83	210,261	0,21796719		0,86054604
	SOD 3 NC	48,131	152,937	0,31471129		1,24249685
	SOD 3 C	170,525	207,642	0,82124522		3,24231968
	SC					
	WT 4 NC	155,632	196,25	0,7930293		1
	WT 4 C	175,267	196,445	0,89219374		1,12504512
	SOD 4 NC	214,574	168,424	1,27401083	FOLD	1,60651168
	SOD 4 C	198,655	115,816	1,71526387		2,16292622
Gel 4	SC					

A$_{2A}$R CTX+ SC caffeine gel 5

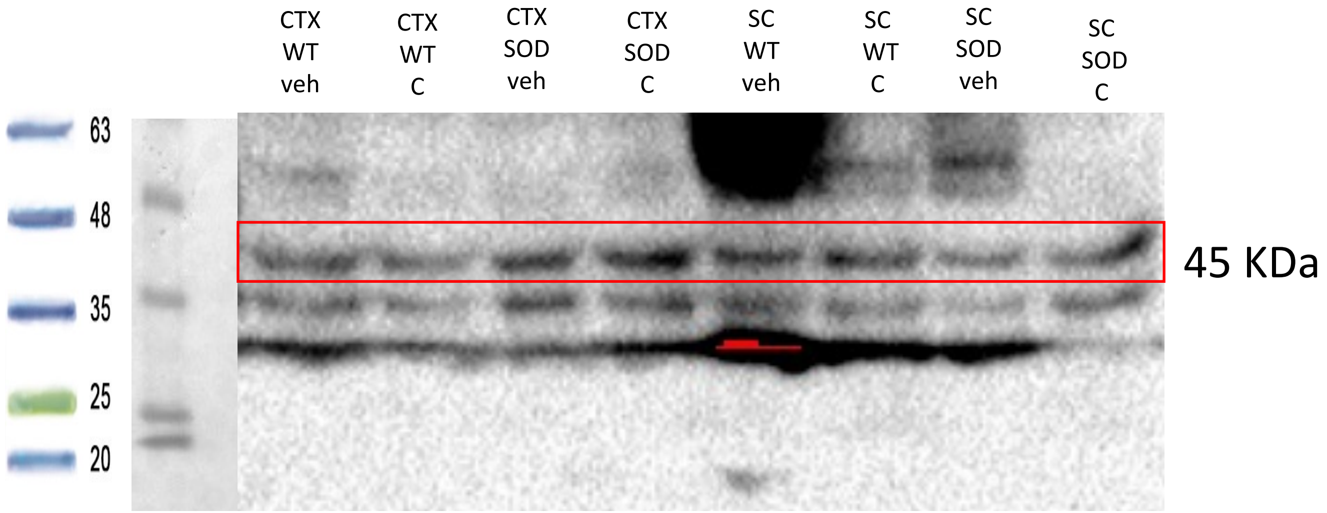

GAPDH CTX + SC caffeine gel 5

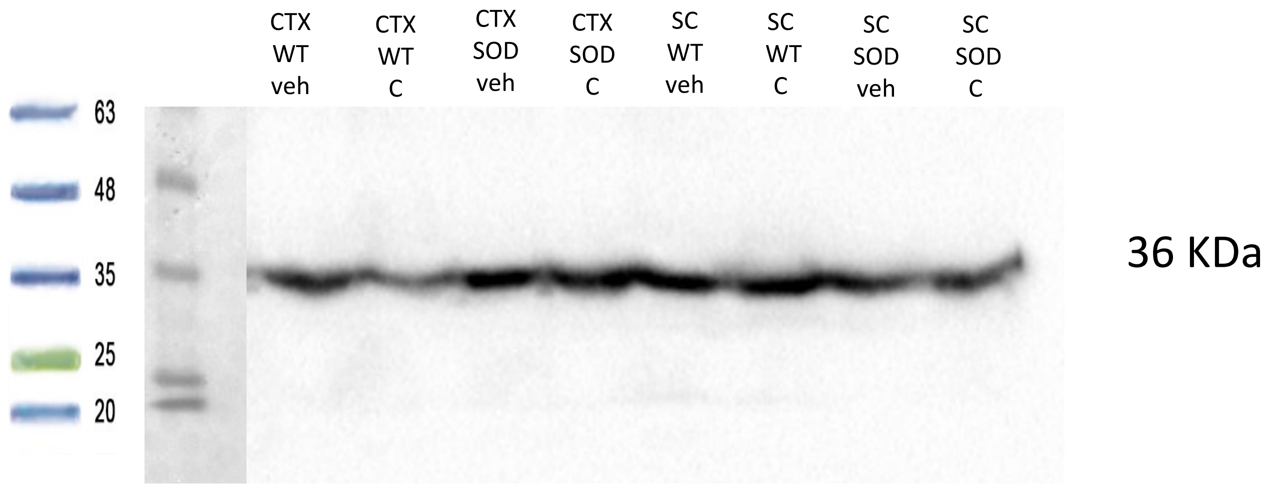

GAPDH is the same for A1R and A2AR caffeine (stripping Western blot membranes)

Densitometry values $A_{2A}R$/GAPDH gel 5

		A2AR	GAPDH	A2AR/GAPDH		A2AR
	WT 5 NC	153,25	152,54	1,00465452		1
	WT 5 C	123,182	117,972	1,04416302		1,039325
	SOD 5 NC	180,573	186,226	0,96964441		0,965152
	SOD 5 C	220,583	182,641	1,20774087		1,202145
	CTX					
	WT 5 NC	208,005	179,415	1,15935122		1
	WT 5 C	183,01	187,453	0,97629806		0,842107
	SOD 5 NC	121,35	124,545	0,97434662	FOLD	0,840424
	SOD 5 C	156,247	99,173	1,57549938		1,358949
Gel 5	SC					

Gel 5 (2nd part of the gel) – Spinal cord (SC)_Figure 7D

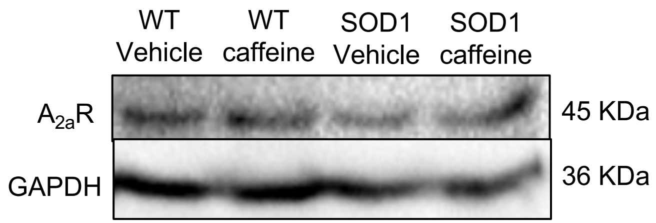

Supplement: S1 Raw images — (PDF) [file pone.0272104.s003.pdf]
